# Embedding-theory-based simulations using experimental electron densities for the environment

**DOI:** 10.1107/S2053273320008062

**Published:** 2020-07-20

**Authors:** Niccolò Ricardi, Michelle Ernst, Piero Macchi, Tomasz Adam Wesolowski

**Affiliations:** a Department of Physical Chemistry, University of Geneva, 30, Quai Ernest-Ansermet, CH-1211 Genève 4, Switzerland; b University of Bern, Freiestraße 3, 3012 Bern, Switzerland; c Department of Chemistry, Materials and Chemical Engineering, Polytechnic of Milan, via Mancinelli 7, Milano 20131, Italy

**Keywords:** quantum crystallography, density embedding, multi-scale simulations, electronic structure, chromophores

## Abstract

The basic idea of frozen-density embedding theory (FDET) is the constrained minimization of the Hohenberg–Kohn density functional *E* ^HK^[ρ] performed using the auxiliary functional , where Ψ_*A*_ is the embedded *N*_*A*_-electron wavefunction and ρ_*B*_(**r**) is a non-negative function in real space integrating to a given number of electrons *N*_*B*_. This choice of independent variables in the total energy functional  makes it possible to treat the corresponding two components of the total density using different methods in multi-level simulations. The application of FDET using ρ_*B*_(**r**) reconstructed from X-ray diffraction data for a molecular crystal is demonstrated for the first time. For eight hydrogen-bonded clusters involving a chromophore (represented as Ψ_*A*_) and the glycylglycine molecule [represented as ρ_*B*_(**r**)], FDET is used to derive excitation energies. It is shown that experimental densities are suitable for use as ρ_*B*_(**r**) in FDET-based simulations.

## Abstract 2

For the first time, the use of experimentally derived molecular electron densities as ρ_*B*_(**r**) in calculations based on frozen-density embedding theory (FDET) of environment-induced shifts of electronic excitations for chromophores in clusters is demonstrated. ρ_*B*_(**r**) was derived from X-ray restrained molecular wavefunctions of glycylglycine to obtain environment densities for simulating electronic excitations in clusters.

## 1. Introduction   

Frozen-density embedding theory (FDET) is the Hohenberg–Kohn theorems-based formal framework for multi-level simulations (Wesolowski, 2004 ▸). The total electron density is built up from two components, ρ_*A*_(**r**) and ρ_*B*_(**r**), of which only the first is constructed from quantum-mechanical descriptors. FDET was originally formulated for variational methods used to obtain such descriptors of the embedded species as: (i) a non-interacting reference system described with a Kohn–Sham determinant (Wesolowski & Warshel, 1993 ▸), (ii) an interacting system described with a multi-determinant wavefunction (Wesołowski, 2008 ▸), and (iii) a one-particle density matrix (Pernal & Wesolowski, 2009 ▸). An extension of FDET for non-variational methods has recently been formulated (Zech *et al.*, 2019 ▸). Extensions of FDET for excited states can be made based on the response theory for either non-interacting (Wesolowski, 2004 ▸) or interacting (Höfener *et al.*, 2012 ▸) systems. Another possibility for describing excited states relies on the Perdew–Levy theorem on extrema of the ground-state energy functional (Perdew & Levy, 1985 ▸). It makes it possible to interpret other-than-the-lowest-energy stationary embedded wavefunctions obtained in FDET as excited states, as pointed out by Khait & Hoffmann (2010 ▸). In any of these variants of FDET, the embedded wavefunction depends on the chosen ρ_*B*_(**r**). Several computational methods sharing some elements with FDET but differing in some key aspects, such as the choice of independent variables, self-consistency between the embedding potential and the embedded wavefunction, locality of the embedding potential *etc.*, have been developed by various groups. We address the reader to reviews concerning – besides the methods based on FDET – related computational approaches (Wang & Carter, 2000 ▸; Wesolowski, 2006 ▸; Jacob & Neugebauer, 2014 ▸; Wesolowski *et al.*, 2015 ▸; Krishtal *et al.*, 2015 ▸).

At the present state of development of approximations for the FDET embedding functional [see equation (8) below], applications of FDET are limited to such systems where ρ_*A*_(**r**) and ρ_*B*_(**r**) do not overlap significantly (Wesolowski *et al.*, 1996 ▸; Bernard *et al.*, 2008 ▸). As a rule of thumb, FDET-based methods are only applicable to such cases where the environment is not covalently bound to the embedded species (Götz *et al.*, 2009 ▸; Goodpaster *et al.*, 2010 ▸; Fux *et al.*, 2010 ▸). In such cases, the overlap between ρ_*A*_(**r**) and ρ_*B*_(**r**) is small and simple local and semi-local approximations are sufficiently accurate. FDET-based simulations can be seen as a variant of quantum mechanics/molecular mechanics simulations, in which the modeller decides on the procedure to generate ρ_*B*_(**r**) instead of parametrizing the force-field parameters describing the energy contributions due to the interactions between the quantum system and its environment. Various system- and property-specific protocols for generating ρ_*B*_(**r**) for FDET-based simulations are possible. Some examples of different treatments of the environment density are given below. If the environment comprises several weakly bound molecules, the corresponding ρ_*B*_(**r**) can be obtained either from quantum-mechanical calculations for the whole cluster comprising all molecules in the environment or, in a simplified manner, as a superposition of molecular densities derived from some quantum-mechanical method (Wesolowski & Warshel, 1994 ▸; Humbert-Droz *et al.*, 2014 ▸). If ρ_*B*_(**r**) is localized in a pre-defined part of space, the effect of electronic polarization of the environment by the embedded species can be taken into account by optimizing ρ_*B*_(**r**) also (Wesolowski & Weber, 1996 ▸) or by ‘pre-polarizing’ it using simpler techniques (Zbiri *et al.*, 2004 ▸; Ricardi *et al.*, 2018 ▸). FDET can also be used to set up a multi-physics simulation in which ρ_*B*_(**r**) represents a statistical ensemble-averaged electron density [〈ρ_*B*_〉(**r**)] represented as a continuum derived using classical statistical-mechanics-based approaches (Kaminski *et al.*, 2010 ▸; Laktionov *et al.*, 2016 ▸). Such methods are especially useful for studying the electronic structure of solvated molecules (Shedge *et al.*, 2014 ▸).

The above examples show clearly that the choice of the procedure to generate ρ_*B*_(**r**) is the key element of any FDET-based simulation. This can be made in an ‘automatic’ way by making some system-independent procedures, choices or approximations, or made in a system-dependent manner involving user-provided information about ρ_*B*_(**r**) such as: (i) using as ρ_*B*_(**r**) the ground-state density of some system obtained without putting in any information about the embedded species, (ii) localizing ρ_*B*_(**r**) in a pre-defined region of space by choosing a limited set of atom-centred basis functions, (iii) allowing it to spread over the whole system, (iv) optimizing ρ_*B*_(**r**) by means of the ‘freeze-and-thaw’ minimization of the total energy (Wesolowski & Weber, 1996 ▸), or (v) any combination of the above. In principle, the density ρ_*B*_(**r**) obtained from the unique partitioning of the total density using the approach developed by Carter and collaborators (Huang *et al.*, 2011 ▸; Huang & Carter, 2011 ▸) could be used as a possible ‘automatic’ procedure to generate ρ_*B*_(**r**) in FDET.

The strategy by which ρ_*B*_(**r**) is obtained from quantum-mechanical calculations for the environment only, *i.e.* in the absence of the embedded species, is particularly attractive. Both our experience and work by other researchers show that obtaining ρ_*B*_(**r**) from an isolated calculation yields the dominant contribution to the complexation-induced shifts of the excitation energies, especially for excitations with shifts of large magnitude [see Fradelos *et al.* (2011 ▸), Zech *et al.* (2018 ▸) and Ricardi *et al.* (2018 ▸)]. Daday and co-workers showed that the effects of the optimization of ρ_*B*_(**r**), either for the ground state alone or for both ground and excited state, are secondary, albeit numerically non-negligible: the excitation energy for methyl­ene­cyclo­propene solvated by 17 water molecules (for which the reference shift obtained from the reference calculations for the whole cluster equals 0.86 eV) is fairly well reproduced (0.82 eV) with such a choice for ρ_*B*_(**r**) (Daday *et al.*, 2014 ▸). In the case of *n*–π* excitations for acrolein in water, the corresponding shifts are 1.42 and 1.10 eV, respectively. Although the difference between the FDET with such a choice of ρ_*B*_(**r**) and the reference shifts cannot be attributed to the ‘neglect of the electronic polarization of the environment’ within the formal framework of FDET, the optimization of ρ_*B*_(**r**) (Daday *et al.*, 2014 ▸) or pre-polarization (Ricardi *et al.*, 2018 ▸) usually reduces this difference. This secondary importance of the explicit treatment of the polarization of the environment is due to the variational character of FDET and the fact that the partitioning of the total density of the complex into ρ_*A*_(**r**) and ρ_*B*_(**r**) is not unique in exact FDET, resulting in a better capacity to approach the exact total density [see the discussions by Wesolowski *et al.* (2015 ▸) and Humbert-Droz *et al.* (2014 ▸)].

The present work concerns yet another possibility for generating ρ_*B*_(**r**) for FDET simulations of embedded species in a given environment consisting of non-covalently bound molecules, in which ρ_*B*_(**r**) is obtained from experimental data concerning a different system: a molecular crystal of the environment molecule. Recent years have brought a number of reports showing that both electron densities (Hansen & Coppens, 1978 ▸) and wavefunctions (Jayatilaka, 2012 ▸) can be reconstructed from X-ray diffraction data. It is tempting, therefore, to explore these new possibilities to generate ρ_*B*_(**r**) for use in FDET-based simulations. We have to emphasize that several approximations may undermine the use of X-ray-based densities for FDET. The most important issues can be listed as follows: (i) the link to any experimental quantity is of course affected by experimental errors, which are unavoidable and may affect both precision and accuracy; (ii) the electron density and wavefunction that are extracted from experiment are static, whereas atoms are not steady in the crystal; (iii) the sampling of the diffraction in reciprocal space is necessarily incomplete; (iv) only the intensity of the diffracted ray is measured, and not the phase; and (v) the crystal sample is imperfect. For these reasons, the possibility of using experimental densities as ρ_*B*_(**r**) in FDET hinges critically on robust and numerically stable protocols to generate such densities, and it is thus important to investigate the dependence of FDET results on such procedures. The current state of the art in wavefunction and electron-density reconstruction from X-ray diffraction data encourages this attempt. In particular, the above-mentioned pitfalls may be tackled as follows: (i) modern instrumentation enables measurement of the diffraction intensities with high precision; (ii) the deconvolution of thermal motion is reliable if the measurements are carried out at a sufficiently low temperature and if the resolution of the diffraction is sufficiently large; (iii) complementary input from theory can compensate for the missing information; (iv) appropriate modelling enables the phasing of the diffracted rays; and (v) data correction from the ideal kinematic theory of diffraction allows for sufficiently accurate data. The present work reports an exploratory study of the use of densities from X-ray restricted wavefunctions in FDET.

Concerning a particular variant of FDET and system to be investigated, we have chosen to evaluate the excitation energies obtained from LinearizedFDET (Wesolowski, 2014 ▸; Zech *et al.*, 2015 ▸) for several organic chromophores, each hydrogen-bonded to its environment. Our extensive benchmarking of the performance of FDET for such cases indicates that the errors in FDET excitation energies due to the approximations used for the explicit density functional for non-electrostatic components of the FDET embedding potential (see the next section) are small. In a benchmark set of embedded organic chromophores, the average deviation from the reference amounts to about 0.04 eV (Zech *et al.*, 2018 ▸). This magnitude of the deviation defines the threshold for complexation-induced shifts in the excitation energy above which analysis of the dependence of the shift on ρ_*B*_(**r**) is meaningful. In the embedded chromophores chosen for the present study, these shifts vary between 0.15 and 0.6 eV.

## 2. Embedded chromophores   

**Figure 1:**
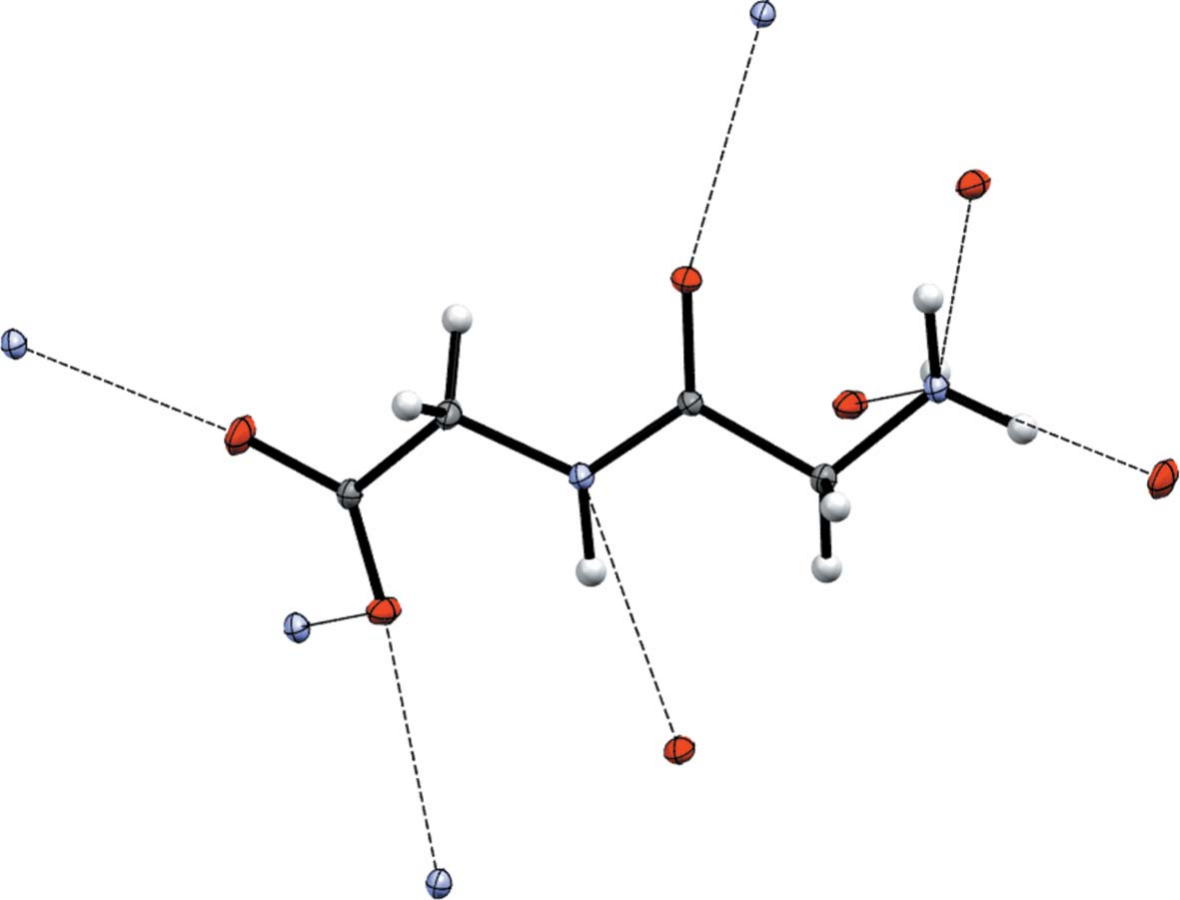
The hydrogen-bonding pattern (dashed lines) for the glycylglycine molecule in the crystal, taken from Dos Santos *et al.* (2014 ▸). Only nearest atoms involved in hydrogen bonding are shown: oxygen (red) and nitrogen (blue).

Concerning the molecules for which ρ_*B*_(**r**) is generated, we have chosen glycylglycine (GlyGly). For this exploratory study, it is crucial that the molecule(s) corresponding to ρ_*B*_(**r**) are capable of forming hydrogen bonds with the chromophore. GlyGly satisfies this condition. Moreover, the molecular density of GlyGly reconstructed from X-ray diffraction data reflects features arising from intermolecular hydrogen bonds present in the crystal structure (Genoni *et al.*, 2018 ▸). Fig. 1 ▸ shows the GlyGly molecule, together with its nearest neighbours in the crystal.

**Figure 2:**
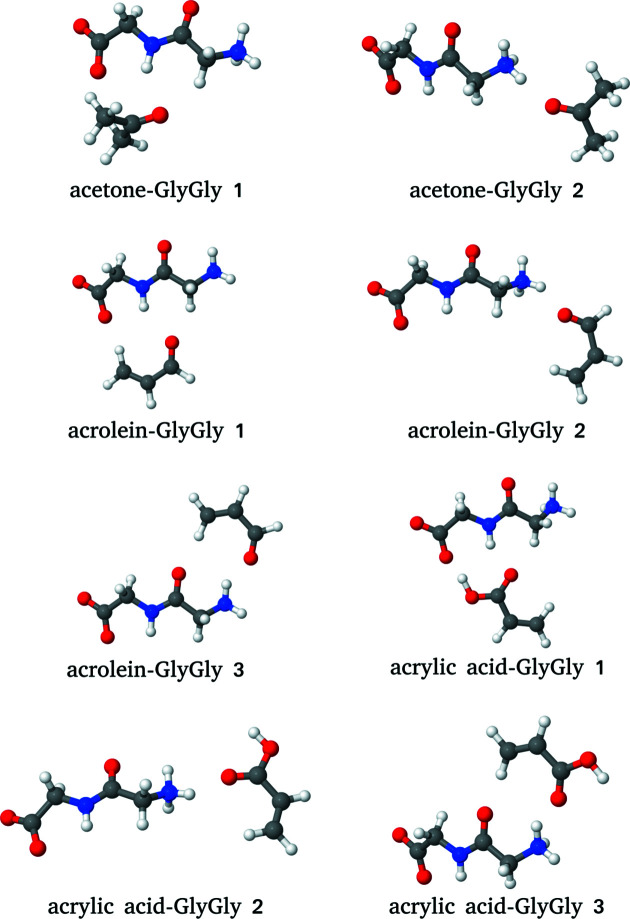
The complexes of three different chromophores with glycylglycine.

The densities reconstructed from experimental data on glycylglycine are used in the present work as ρ_*B*_(**r**) in FDET calculations of excitation energies for eight different hydrogen-bonded complexes formed by one organic chromo­phore (acrolein, acrylic acid or acetone) and glycylglycine. Fig. 2 ▸ shows the considered clusters.

The hydrogen-bonding networks shown in Figs. 1 ▸ and 2 ▸ are not the same. In the crystal, all donors and acceptors are involved in hydrogen bonding, which is not the case in the investigated clusters. Nevertheless, each individual hydrogen bond in the clusters has its corresponding partner in the crystal. It can be expected, therefore, that the effect of the hydrogen bonding on ρ_*B*_(**r**) in the cluster is also reflected in the density obtained from the crystal.

## 3. FDET approach to multi-level simulations   

For a system comprising *N_AB_* electrons in an external potential *v*_*AB*_(**r**), the functional 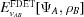 is defined to satisfy by construction the following relation:
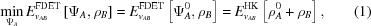
where 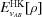 is the Hohenberg–Kohn ground-state energy functional (Hohenberg & Kohn, 1964 ▸) and 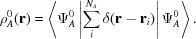



By virtue of the second Hohenberg–Kohn theorem, equation (1) leads to
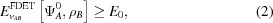
where 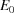 = 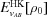 and ρ_0_(**r**) are the ground-state energy and density of the total system, respectively. Equality is reached for a large class of densities ρ_*B*_(**r**), 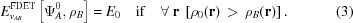
Using conventional density functionals representing components of the total energy, and arbitrary partitioning of the external potential *v*_*AB*_(**r**) = *v*_*A*_(**r**) + *v*_*B*_(**r**) leads to a form of 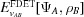 more suitable for further discussions,
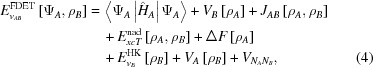
where
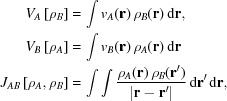
and 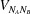 is the interaction energy between the nuclei defining *v*_*A*_(**r**) and *v*_*B*_(**r**). The non-additive bi-functional 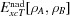 is related to the functionals *E*_*xc*_[ρ] and *T*_*s*_[ρ] defined in the constrained-search formulation of the Kohn–Sham formalism (Levy, 1979 ▸). It is defined as
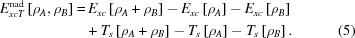
The functional Δ*F*[ρ], on the other hand, depends on the form of the wavefunction Ψ used in equation (1) and is also defined via the constrained search (Wesołowski, 2008 ▸). For instance, if Ψ_*A*_ is a single determinant (Φ), it reads
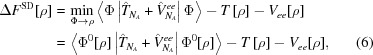
where 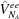 is the electron–electron repulsion operator, 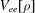 is the density functional of the electron–electron repulsion energy, and 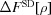 is just the correlation functional (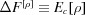) defined in the constrained-search formulation of density functional theory (Levy, 1979 ▸; Baroni & Tuncel, 1983 ▸). For Ψ of the full CI form, Δ*F* ^FCI^[ρ] = 0 by definition.

Euler–Lagrange optimization of Ψ_*A*_ leads to the Schrödinger-like equation
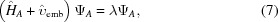
where
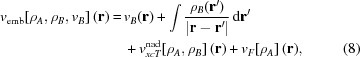
with 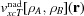 and *v*_*F*_[ρ_*A*_](**r**) being the first functional derivatives of 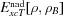 and Δ*F*[ρ], respectively.

The lowest-energy solution of equation (7) will be denoted as 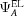. Note that the energy is given not by the Lagrange multiplier λ but by equation (4). For exact density functionals, any variational method can be used to obtain 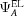 and the corresponding density 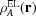, which satisfy by construction the basic FDET equality given in equation (1).

### 3.1. Reconstruction of ρ_*B*_(**r**) from X-ray diffraction data   

X-ray restrained wavefunctions (XRW), in the literature commonly (but incorrectly) termed X-ray constrained wavefunctions, were initially developed by Jayatilaka and co-workers (Jayatilaka, 2012 ▸; Jayatilaka & Grimwood, 2001 ▸; Grimwood & Jayatilaka, 2001 ▸). Within the XRW paradigm, instead of applying the variational principle, like in conventional SCF, a special functional *L* is defined, based on a classical Hamiltonian and a function of the square difference between calculated and experimentally measured structure factors, which is ideally distributed with χ^2^ statistics. Here, 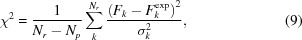
with *N*_*r*_ and *N*_*p*_ being, respectively, the number of experimental data and the number of parameters in the model. 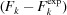 is the difference between the structure factors from the trial wavefunction and the experimental ones, and σ_*k*_ is the experimental standard deviation. Thus, the minimization of *L* implies finding the minimal energy AND the best agreement with experiment. Of course, these cannot be achieved simultaneously and a parameter λ_*J*_ must be defined in order to weight the two parts of the functional. Therefore, the functional takes the form
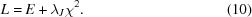
This procedure allows the construction of molecular wavefunctions from experimental observations in crystals. By increasing λ_*J*_, both long- and short-range interactions in the crystal are progressively taken into account. In this work, we used structure factors measured for GlyGly to calculate X-ray restrained wavefunctions with λ_*J*_ values from 0.0 to 1.0, since for higher values the SCF procedure does not converge. We stress that such a value of λ_*J*_ = 1.0 has no specific meaning because the electronic energy of the Hamiltonian and the electron-density difference in the χ^2^ function have two different units; thus λ_*J*_ is not dimensionless but depends on the number of electrons, the molecular volume and the diffraction resolution. Moreover, the structure factors in the χ^2^ function are weighted by the variance of their measurement statistics. The aforementioned wavefunctions were then used to calculate ρ_*B*_(**r**).

### 3.2. Computational details   

The following approximations were used in the reported FDET calculations: (i) ADC(2) treatment (Schirmer, 1982 ▸) of correlation for embedded *N*_*A*_ electrons as implemented by Prager *et al.* (2016 ▸), (ii) decomposable approximations for 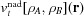 (LDA) and 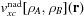 (note that in the LinearizedFDET used here, approximations for the energy components 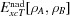 and Δ*F*[ρ_*A*_] are not used at all), (iii) neglect of the *v*_*F*_[ρ_*A*_] contribution to the embedding potential, (iv) monomer expansion of ρ_*A*_(**r**) (only atomic basis sets centred on the chromophore), (v) monomer expansion of ρ_*B*_(**r**) (only atomic basis sets centred on GlyGly), and (vi) chromophore-independent generation of ρ_*B*_(**r**) using one of the following methods for the isolated GlyGly: Hartree–Fock, first-order Møller–Plesset (MP) perturbation theory, Kohn–Sham (KS) with PBE (Perdew *et al.*, 1996 ▸) approximation for *E*_*xc*_[ρ], coupled clusters singles and doubles (CCSD) or the reconstruction from experimental structure factors (see the next section).

The FDET results for each cluster are compared with the reference obtained from ADC(2) calculations. The reported reference shifts in the excitation energy are evaluated as 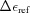 = 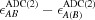, where *AB* denotes the complex and *A*(*B*) denotes the chromophore alone but with the basis set expanded by the functions localized on GlyGly [similar to how it is done in the counterpoise technique of Boys & Bernardi (1970 ▸) for intermolecular interaction energy]. In all calculations, including also the reconstruction of the electron density of GlyGly from the X-ray structure factors, the 6-311G basis set was used.

At λ_*J*_ = 0, the wavefunction obtained from the X-ray diffraction data is just the Hartree–Fock molecular wave­function. The numerical results should be identical, regardless of what software is used to generate ρ_*B*_(**r**). We used this fact to check the numerical soundness of the procedures to export–import densities ρ_*B*_(**r**) obtained with different software. *Tonto* (Grimwood *et al.*, 2003 ▸) was used for X-ray restrained wavefunction calculations, *Psi-4* (Parrish *et al.*, 2017 ▸) to generate the CCSD GlyGly density, and *Q-Chem* (Shao *et al.*, 2015 ▸), with its *ADCMAN* (Wormit *et al.*, 2014 ▸) and *FDEMAN* (Prager *et al.*, 2016 ▸) modules, for all other calculations, including FDET/ADC(2) ones.

Throughout this article, 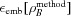 (and 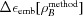) denote the FDET-derived excitation energy (and environment-induced shift), where the superscript in 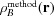 specifies the method used to generate ρ_*B*_(**r**).

## 4. Results   

**Figure 3:**
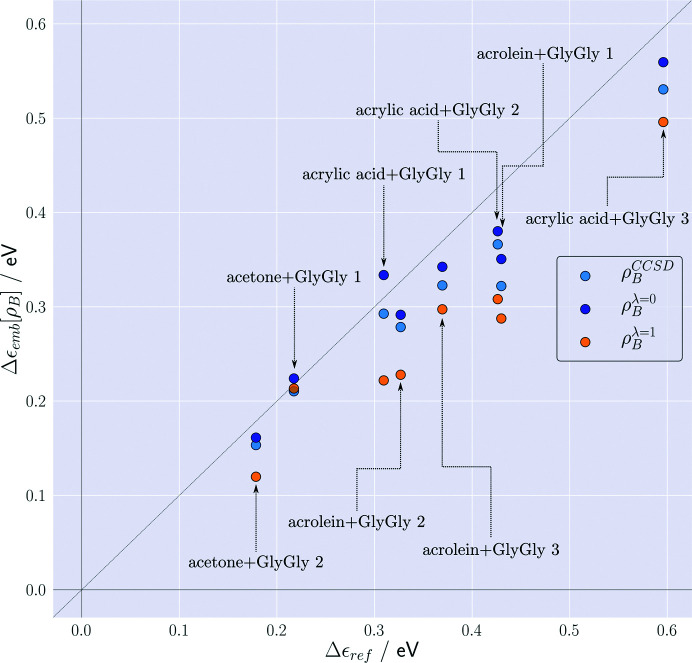
Complexation-induced shifts of the excitation energy (Δ∊_emb_[ρ_*B*_]) for eight chromophores hydrogen-bonded to GlyGly. For each complex, FDET calculations are made [embedded ADC(2)] using as ρ_*B*_(**r**) the electron density of GlyGly obtained from three different methods: Hartree–Fock (λ_*J*_ = 0) or CCSD for the isolated GlyGly, or density reconstructed from X-ray structure factors for the GlyGly molecular crystal at λ_*J*_ = 1. Reference values (Δ∊^ref^) are obtained from ADC(2) calculations for the whole complex.

For the eight considered clusters, the lowest excitation energies obtained from FDET/ADC(2) calculations (∊_emb_[ρ_*B*_]) using several choices for ρ_*B*_(**r**) are shown in Fig. 3 ▸, together with the corresponding reference supermolecular ADC(2) results. These excitations have *n*–π* character and are blue-shifted due to the interactions with the environment. The magnitude of the reference shift falls in the 0.15–0.6 eV range, which makes the shift in these complexes a suitable observable for discussing the effect of the ρ_*B*_-dependency of the FDET results. For this type of excitation, the combined effect of the approximation used for the FDET embedding potential and the use of the isolated environment density as ρ_*B*_(**r**) results in an average error in the excitation energy of magnitude 0.04 eV (Zech *et al.*, 2018 ▸; Ricardi *et al.*, 2018 ▸).

We start with an analysis of the results obtained without taking any experimental information from the molecular crystal, *i.e.* 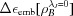. 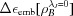 corresponds to a ‘standard’ FDET protocol in which the Hartree–Fock density of the isolated environment is used as ρ_*B*_(**r**). The deviations from the reference are small and their magnitude is consistent with the benchmark results published elsewhere (Zech *et al.*, 2018 ▸). The effect of correlation on ρ_*B*_(**r**) [see the shifts obtained with 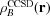] results in a slight reduction of the shifts in all cases.

At λ_*J*_ > 0, both the correlation and the crystal-field effects are taken into account in ρ_*B*_(**r**), leading to a further reduction in the shifts. The deviations of FDET shifts from the reference increase (see the values of 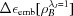 in Fig. 3 ▸).

As previously mentioned, we could not extend the restraint to values of λ_*J*_ larger than 1, because the procedure became numerically unstable (Genoni *et al.*, 2018 ▸). Unfortunately, although the effects of correlation and polarization by the crystal field are reflected in 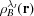, they cannot be separated. Moreover, the environment of GlyGly in the molecular crystal and in the clusters analysed in the present work are different. As a result, even if the reconstruction of the density of GlyGly from X-ray structure factors were exact, this would not guarantee that such density would yield the best FDET results for the clusters under investigation. The values 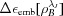 at λ_*J*_ = 0 and λ_*J*_ = 1 represent, therefore, a good estimate of the maximum scatter (minimal and maximal bounds) of the FDET results due to the ρ_*B*_-dependency of the FDET embedding potential. Within these bounds, the deviations from the reference do not exceed 0.1 eV (or 30% in terms of the relative error). This also points out the need for a thorough analysis of the disentangled effects of correlation and polarization in 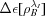.

The subsequent part concerns the numerical stability of the FDET-derived complexation-induced shifts of the lowest excitation energy with respect to variations of ρ_*B*_(**r**) corresponding to the change in the parameter λ_*J*_ from 0 to 1.

**Figure 4:**
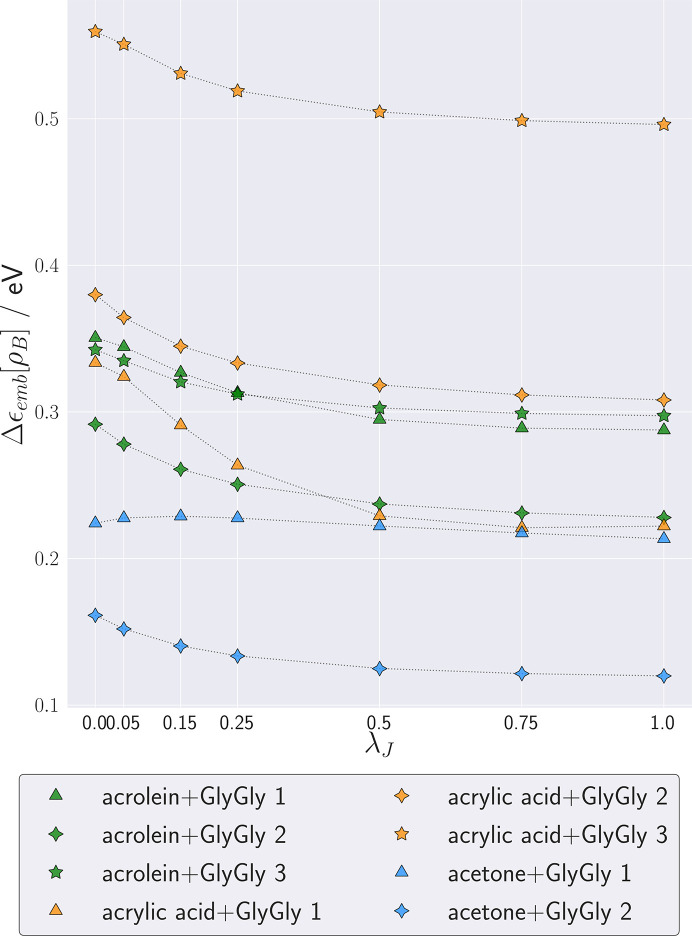
Complexation-induced shifts of the excitation energy (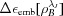) at various values of λ_*J*_ for eight clusters.

Fig. 4 ▸ shows the dependence of the calculated shifts 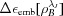 on the parameter λ_*J*_ for each complex. The dependence of 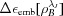 on λ_*J*_ is smooth and monotonic. Above λ_*J*_ = 0.5 up to the maximum value used in this study, λ_*J*_ = 1, 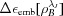 remains almost constant (it changes by as little as about 0.01 eV). The magnitude of the solvatochromic shift decreases for all but one system (acetone + GlyGly 1) when λ_*J*_ increases. This can be ascribed to a decrease in dipole-moment magnitude for the XRW densities when λ_*J*_ increases. A similar trend appears for correlated methods, which, as is known, tend to yield lower dipole moments.

**Figure 5:**
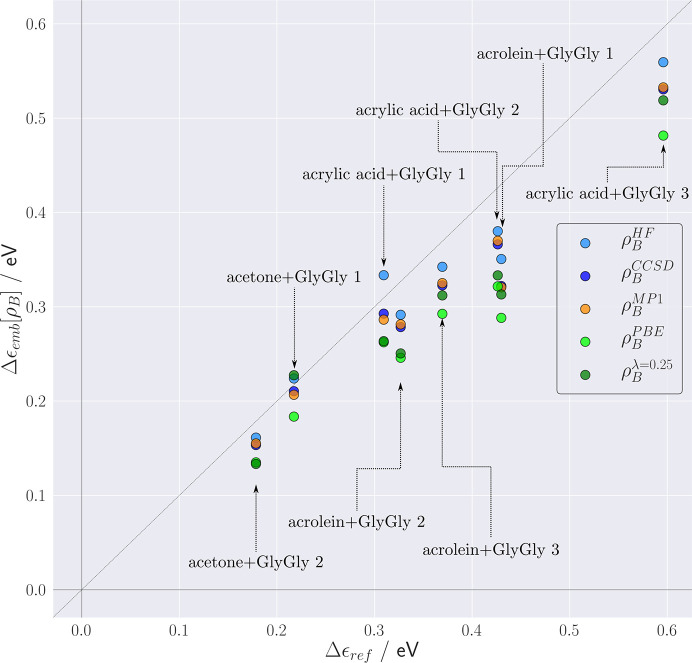
FDET [embedded ADC(2)] derived complexation-induced shifts of the excitation energy (Δ∊_emb_) obtained for eight intermolecular complexes with different choices for ρ_*B*_(**r**). Reference values (Δ∊^ref^) are obtained from ADC(2) calculations for the whole complex.

Turning back to practical applications, we notice that large-scale simulations usually apply the monomer expansion for both ρ_*A*_(**r**) and ρ_*B*_(**r**), and using the isolated environment density as ρ_*B*_(**r**) already assures good accuracy of the FDET-derived environment-induced shifts. In such simulations, the modeller has a wide range of available methods to generate ρ_*B*_(**r**) (see the *Introduction* [Sec sec1]). The data collected in Fig. 5 ▸ show how the FDET results depend on the method used to generate ρ_*B*_(**r**), including Hartree–Fock, MP1, CCSD and KS-DFT(PBE). For reference purposes, the values of Δ∊_emb_ obtained from X-ray diffraction data at λ_*J*_ = 0.25 are also given.

The results collected in Fig. 5 ▸ indicate clearly that X-ray-derived molecular densities are suitable for generating ρ_*B*_(**r**) for FDET calculations following the conventional protocol [LinearizedFDET, monomer expansion of ρ_*A*_(**r**), monomer expansion for ρ_*B*_(**r**), lack of explicit treatment of ρ_*B*_(**r**) polarization by the chromophore]. The deviations from the reference are, however, larger than if Hartree–Fock or correlated isolated GlyGly densities are used for this purpose. This does not bear direct relevance to the quality of these densities. The overall error of the FDET-derived excitation energy results from the balance of the errors in two FDET embedding potentials evaluated at two different pairs of densities, 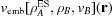 and 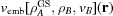, where 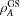 and 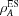 denote the ground and excited state, respectively, in which the non-electrostatic contributions are approximate.

It is worth noting that the use of X-ray-derived densities as ρ_*B*_(**r**) leads to smaller errors than if the Kohn–Sham PBE calculations are used for this purpose.

## 5. Conclusions   

Recent developments in techniques to reconstruct the electron density from X-ray diffraction data have made it possible not only to determine the maxima of the electron density (coinciding with the positions of nuclei) but also to reveal its more detailed features (Genoni *et al.*, 2018 ▸). For a molecular crystal, the reconstruction yields a localized density of a single molecule but taking into account its chemical environment. In FDET-based simulations, the molecular densities are used as an input quantity providing the complete quantum-mechanical descriptor of the environment of the embedded species. In the present work, we explored the possibility of using the mol­ecular density of glycylglycine derived from X-ray diffraction data collected for the molecular crystal in FDET calculations of the complexation-induced shift of the excitation energy in eight intermolecular complexes, each consisting of an organic chromophore hydrogen-bonded to one glycylglycine mol­ecule.

The usability of such densities for this purpose was not evident before the present study was made. Several factors could, in principle, invalidate such practical applications of X-ray reconstructed densities. First of all, glycylglycine in the crystal and in the complexes analysed in the present work has different environments. This might result in different polarization of such localized molecular densities, and as a consequence, contribute to errors in the FDET results. Other factors relate rather to the reconstruction procedure. It cannot be made perfect due to (i) errors in the experimental measurements, (ii) the very basic assumption according to which the average of a dynamic quantity (electron density) is represented using an intermediate object, namely a static single-determinant wavefunction, (iii) incompleteness of the experimental data, (iv) errors in the phasing procedures, and (v) crystal defects. It might be expected, therefore, that the reconstructed densities would indeed not deviate significantly from the constraint-free one and, in turn, yield similar excitation energies if used as ρ_*B*_(**r**). The primary objective of this work was the verification of this expectation. The obtained results demonstrate, indeed, that X-ray reconstructed densities are suitable to be used as ρ_*B*_(**r**) in FDET on a par with possible alternative techniques.

Despite the fact that the X-ray restrained wavefunction procedure does not yield a unique solution, but rather a range of densities parametrized by λ_*J*_, the scatter of the excitation energies obtained using the whole range of this parameter is rather narrow. For all but one complex (acetone + GlyGly 1, characterized by a remarkably short hydrogen bond of only 1.3 Å) the excitation energies vary within approximately 0.05 eV, depending on the details of the reconstruction procedure. This scatter in calculated shifts is small compared with the range of variation in solvatochromic shifts (Reichardt, 1994 ▸; Improta *et al.*, 2016 ▸), making FDET simulations using X-ray-derived molecular densities an attractive tool for making quantitative predictions and interpreting experimental results. Further reduction of this scatter is probably possible through disentangling the effect of crystal-field polarization and the correlation effect on the electron density of a molecule in a molecular crystal. We intend to deal with this issue in our subsequent work. Also here the experimentally derived ρ_*B*_(**r**) might prove more useful than alternative techniques. This is the case when the molecules associated with ρ_*B*_(**r**) have similar neighbours in the cluster to be investigated and in the molecular crystal used to generate ρ_*B*_(**r**).

For the first time, we adopted an experimental density for the environment and tested this on spectral shifts for valence excitations. At present, a density calculated via an X-ray restrained procedure is the only possibility for this approach. The numerical examples in this work in which we applied the proposed procedure concern microsolvated clusters. For finite systems, many alternatives to generate ρ_*B*_(**r**) involving similar or even lower computational cost are possible (Hartree–Fock or Kohn–Sham densities of isolated environments including, or not, their optimization or pre-polarization). This numerical validation is the first stage in our long-standing interests and plans aimed at modelling the electronic structure of species in the condensed phase such as neat or doped molecular crystals (Meirzadeh *et al.*, 2018 ▸). We plan to apply the same strategy to generate the FDET embedding potential using experimentally derived ρ_*B*_(**r**) for modelling other spectroscopic properties (core excitations, NMR shifts, two-photon absorption, hyperpolarizabilities *etc.*) that are evaluated from embedded wavefunctions. In infinite systems, the generation of ρ_*B*_(**r**) from first principles might face serious difficulties if done in a similar way as for the studies of clusters, for the following reasons. Firstly, a density obtained using a straightforward application of the simplest protocol [generation of ρ_*B*_(**r**) in an artificial system with a void in place of the part described by means of Ψ_*A*_] might be unphysical or even impossible to obtain due to convergence problems or the need for a much larger supercell. Secondly, taking into account the effect of electronic correlation on the electron density in periodic systems is generally limited to Kohn–Sham types of methods. A final point is worth discussing. By using a wavefunction restrained to fit the electron density of a molecule in a crystal, the environment density used in the approach we propose here implicitly includes not only the effects of intermolecular interactions but also the long-range electrostatic effects of crystalline matter.
